# The validation of a Mandarin version of the Empathy Components Questionnaire (ECQ-Chinese) in Chinese samples

**DOI:** 10.1371/journal.pone.0275903

**Published:** 2023-01-26

**Authors:** Yabo Ge, Chris Ashwin, Fengying Li, Wei Cao, Yu Zhang, Xuan Zhao, Binghai Sun, Weijian Li

**Affiliations:** 1 Department of Psychology, Zhejiang Normal University, Jinhua, China; 2 Institute of Child Development, Jinhua Polytechnic, Jinhua, China; 3 Centre for Applied Autism Research, Department of Psychology, University of Bath, Bath, United Kingdom; 4 Publicity Department, Zhejiang Normal University, Jinhua, China; 5 College of Mathematics and Computer Science, Zhejiang Normal University, Jinhua, China; Lorestan University, ISLAMIC REPUBLIC OF IRAN

## Abstract

Empathy involves both empathic ability and empathic motivation. An important topic has been how to measure empathic ability and motivation simultaneously in both clinical and non-clinical samples and across different cultures. The Empathy Components Questionnaire (ECQ) is a self-report questionnaire that measures empathic ability and motivation in a questionnaire. The current study aimed to validate the Mandarin Chinese version of the ECQ (ECQ-Chinese) in three Chinese samples. In study 1, a total of 538 Chinese participants (Sample 1) completed the ECQ-Chinese via an online survey, and existing measures of empathy and related constructs which were used for criterion validity. In study 2, a total of 104 participants (Sample 2) were recruited again from sample 1 and completed the ECQ-Chinese three weeks later to investigate test-retest reliability. In study 3, a further 324 participants (Sample 3) completed the ECQ-Chinese for confirmatory factor analysis. The results showed that the ECQ-Chinese has a good internal consistency reliability, split-half reliability, and criterion validity (Study 1), and a good test-retest reliability (Study 2). Further, Study 3 found that a 22-item ECQ-Chinese consisting of five subscales had a good construct validity, convergence validity and discriminate validity, demonstrating it to be a suitable tool for the measurement of empathic ability and motivation in Chinese samples and to carry out cross-cultural studies of empathy and its components.

## Introduction

Empathy research has a long history, beginning with the roots of the term "empathy" emerging within German aesthetics ("*Einfühlung*", which means ‘feeling into’), which was initially proposed by philosopher Robert Vischer in 1873 [1]. However, to date, there is still no general agreement in the field on a comprehensive definition of empathy [2–4]. Most researchers view empathy as involving the ability to understand and share the thoughts and feelings of other people, and to care for their welfare [5–8]. There is also consensus about the involvement of the two key components of cognitive and affective empathy [9–14]. More recent studies have investigated empathic motivation and empathic ability as two further components of empathy, with each component contributing to individual differences in empathy [15–17].

### Empathy: Ability and motivation

Early views regarded empathy and its cognitive and affective components as stable constructs over time [9–14]. Cognitive empathy involves perceiving and inferring another’s mental or emotional state and includes aspects of perspective-taking and mentalizing, although it does not require being in a similar affective state with the other person [14, 18]. Affective empathy encompasses the sharing of another person’s emotional state, but without necessarily understanding about why they are experiencing it [19]. Evidence exists that cognitive and affective empathy are dissociable components, which includes that they have different developmental trajectories [20], involve diverse processes between them [21], and are both reported to have a different neural basis [22].

Although most research has focused on empathic ability, recent studies have highlighted that empathy also involves motivational factors [23–25]. Keysers and Gazzola [26] proposed dissociating the ability versus the propensity for empathy. According to this idea, the ability for empathy is a more stable capacity and represents the upper bound of how strongly a person can engage in a particular facet of empathy. The propensity for empathy refers to empathic motivation, which involves the individual’s tendency towards empathizing as a function of the situation. Furthermore, Zaki [24] considers that empathy is not always automatic but is also context-dependent, which proposes a motivational component. Modifying the subjective value of empathic behavior by reducing the cognitive costs or by increasing the social benefits motivates individuals toward greater empathy [27, 28]. While evidence supports the ideas about dissociable ability and motivational components to empathy, it has proved very difficult to simultaneously measure both of these components, as well as cognitive and affective empathy.

#### Empathy Components Questionnaire (ECQ)

Although various tools have been developed to measure empathy, most have been developed with the purpose to measure empathic ability. Davis [29, 30] developed the widely used Interpersonal Reactivity Index (IRI) questionnaire for measuring empathy as a multidimensional construct, including cognitive and affective empathy. The IRI includes the four subscales of empathic concern, perspective taking, fantasy, and personal distress, and has been widely used in social and personality psychology [31, 32]. Similarly, Lang et al. [33] developed the Multifaceted Empathy Test (MET) to assess an individual’s empathy, which consists of 40 images showing people expressing different emotions (20 images of positive emotions and negative emotions). Participants are required to answer about which feeling they think the person in the picture is experiencing, with only one of four options being correct. A further self-report questionnaire is the Empathy Quotient (EQ), which provides a general measure of empathy [10]. However, none of these tools measures all of the proposed components of empathy, including cognitive and affective empathy as well as both the ability and motivation for empathy. Towards this, Batchelder et al. [34] were the first to develop and validate the Empathy Components Questionnaire (ECQ), which is a valid and reliable self-report tool to measure all the proposed components of empathy.

The original Empathy Components Questionnaire (ECQ) consists of 27 items to measure empathy as a multidimensional construct encompassing both ability and drive components with each domain of affective and cognitive empathy [34]. Each item is rated on a 4-point Likert scale and fourteen of the items are reverse scored. Batchelder et al. [34] explored the factor structure of the ECQ (principal component analysis, PCA) in a group of British college undergraduates (*N* = 101, *M*_age_ = 20.31years, *SD* = 1.90) and validated the ECQ (confirmatory factor analysis and reliability analysis) in another group of British college undergraduates (*N* = 211, *M*_age_ = 27.75years, *SD* = 8.75). The results from the principal component analysis revealed the ECQ had a model consisting of the five factors of cognitive ability (CA) (i.e., the skill, capacity, or potential in perspective-taking and adopting another’s point of view), cognitive drive (CD) (i.e., the motivating interest or tendency in perspective-taking and towards adopting another’s point of view), affective ability (AA) (i.e., the skill, capacity, or potential in recognizing, being sensitive to, and sharing others’ emotional experiences), affective drive (AD) (i.e., the motivating interest or tendency towards recognizing, being sensitive to, and sharing others’ emotional experiences), and affective reactivity (AR) (i.e., to be action-specific by individuals responding to another’s emotional experiences, which often entails sharing these emotions and feelings). The results of a further confirmatory factor analysis demonstrated that the five-factor solution provided a good fit, and Cronbach alpha values for all subscales were acceptable, which suggested that the ECQ has good reliability and validity for concurrently measuring empathic ability and motivation [34].

### Cross-cultural empathy research

There has been limited research to date investigating cultural differences in empathy between Chinese and Western samples, and previous research findings have reported inconsistent results to each other [35–40]. For example, Zhao et al. [39] compared Australian Caucasians and Mainland Chinese samples using the EQ and IRI, and found Culture-Sex interactions such that females differed between the two samples in their empathy scores (with Australian females having higher empathy scores than the Chinese females), but the males did not show differences between samples. In another study, Melchers et al. [36] investigated empathy differences between cultures using the EQ and the IRI comparing samples from mainland Chinese, Germany, Spain, and the United States, and did not report any distinct pattern of cultural differences in empathy between cultures. Some studies have reported differences between Western and Chinese samples on the subscales of the IRI [38, 41, 42], but other studies have reported no differences between samples on the IRI subscales [37, 43, 44]. Similarly, the specific empathy subscales of the IRI reported to differ between cultural samples have varied between studies, showing inconsistent results to date about empathy differences between Western and Chinese samples [45].

Two possible explanations may account for these inconsistent results. Firstly, although the IRI has been widely used to measure empathy, its subscales are not well tied to the components of empathy and they actually measure broader processes than just empathy [10]. For example, the fantasy subscale was designed to evaluate a person’s imagination to appreciate the emotions of fictitious characters in movies, plays, or books (i.e., "When I watch a good movie, I can very easily put myself in the place of a leading character", "I daydream and fantasize, with some regularity, about things that might happen to me"). Some of the items in the personal distress subscale (i.e., "In emergency situations, I feel apprehensive and ill at ease.") assess emotional self-control rather than empathy.

Secondly, empathy is widely regarded as an umbrella term [13, 46]. For example, Gerace [46] believed that empathy covers a range of components, including feeling the same feeling, perspective-taking, personal distress, and propensity to experience empathy. However, none of the previous empathy measures (e.g., the IRI, EQ, and MET) index most of the components of empathy in current theoretical frameworks, including both cognitive and affective empathy as well as ability and motivation within each of these components, to provide a more comprehensive assessment of empathy. The ECQ represents the first published instrument to measure both empathic ability and motivation from the perspective of both cognitive and affective domains, as well as affective reactivity, within the same measure to provide a fast and convenient tool for assessing the multi-dimension nature of empathy. However, to date, the ECQ has only been validated in English and is not currently available for testing samples who speak languages other than English, such as those who speak Chinese languages. The development of a Chinese language version of the ECQ would allow for investigations of empathy and its components in Chinese samples and for carrying out cross-cultural studies comparing empathy and its components between Western and Chinese samples.

### The current study

The current study aimed to validate a Chinese version of the ECQ (ECQ-Chinese) translated into Mandarin and tested in three Chinese samples, to provide a validated measure of empathy and its components in China. It was expected the results from an EFA and CFA with the ECQ-Chinese would demonstrate the same 5-factor model as the original ECQ, and show good criterion validity as tested using other similar measures.

## Study 1: Item analysis and exploratory factor analysis

### Materials and methods

#### Participants

The recruitment advertisements were distributed and collected online through multiple social platforms (i.e., Wechat and Tencent QQ), which contained information about the project, requirements of the survey, the time required, and so on. All the participants in the questionnaire survey were volunteers. A total of 559 undergraduate students were recruited from Zhejiang Normal University, Zhejiang Sci-tech University, and Jinhua Polytechnic in Zhejiang province. This sample (Sample 1) was used to explore the factor structure of the ECQ-Chinese and item analysis to quantitatively determine whether each item should be eliminated or retained. Within this sample, 21 participants were excluded from the data screening process because they chose the same option on all the scales, showing they were not properly engaged in completing the questionnaire. The final sample included 538 undergraduates (84.1% female), with degree topics covering a wide range of areas including pedagogy, science, literature, engineering, philosophy, etc. The sample had a mean age of 20.34 years with an *SD* ± 2.08 years.

#### Measures

To assess the content validity of ECQ-Chinese, several instruments were selected. The rationale for selecting these instruments was as follows. Firstly, it is broadly regarded that empathy is a critical component in interpersonal processes [47, 48], and provides a drive for our prosocial behaviors [2, 49]. Thus, the Prosocial Tendencies Measure (PTM) was selected as the criteria questionnaires. Secondly, a measure of self-esteem was often used in validity analyses of empathy measures [29, 31], and thus we selected Self-Esteem Scale (SES) as a criteria questionnaire. Thirdly, empathy plays a key role in maintaining meaningful social interactions and facilitating relationship satisfaction [50, 51]. Thus, we selected the Basic Psychological Needs Scale (BPNS) as a criteria questionnaire. Finally, the Interpersonal Reactivity Index (IRI) is probably the most widely used instrument to assess individuals’ empathy [52]. Therefore, we include the IRI, the SES, the BPNS, and the PTM as the criteria questionnaires in Study 1.

*Demographic information questionnaire*. The demographic characteristics of the participants were evaluated using a questionnaire that included the following information: gender, age, and major (i.e., "what is your major?")

*Empathy Components Questionnaire (ECQ)*. The ECQ is a 27-item self-report questionnaire consisting of five subscales (CA, CD, AA, AD, and AR) (see *Appendix A in S1 Appendix*). The overall validation processes followed the guidelines for cross-culture adaptation of self-report measures [53]. The original English version of the ECQ was first translated into Mandarin Chinese by an English-Chinese bilingual researcher, and then another three English-Chinese bilingual researchers proofread it. The translated version of the ECQ was then back-translated into English and the original author checked to verify the wording was accurate. The final translation was approved by all members of the expert panel (e.g., psychologists and professional translators).

The ECQ includes five different subscales. Cognitive ability, which is the skill, capacity, or potential in perspective-taking and adopting another’s point of view (6 items, e.g., "I am usually successful in judging if someone says one thing but means another."). Cognitive drive reflects the motivating interest or tendency in perspective-taking and towards adopting another’s point of view (5 items, e.g., "I strive to see how it would feel to be in someone else’s situation before criticizing them."). Affective ability refers to the skill, capacity, or potential in recognizing, being sensitive to, and sharing others’ emotional experiences (5 items, e.g., "I am good at responding to other people’s feelings."). Affective drive involves the motivating interest or tendency towards recognizing, being sensitive to, and sharing others’ emotional experiences (4 items, e.g., "I am not interested in protecting others, even if I know they are being lied to."). Affective reactivity is argued to be action-specific by individuals responding to another’s emotional experiences, which often entails sharing these emotions and feelings (7 items, e.g., "When someone seems upset, I am usually uninterested and unaffected by their emotions."). Responses ranged from 1 (*strongly disagree*) to 4 (*strongly agree*).

*Empathy*. The empathy was measured by Interpersonal Reactivity Index (IRI). The IRI consists of 28 items and assesses an individual’s empathy based on four subscales including; perspective taking (PT), fantasy (FS), personal distress (PD), and empathic concern (EC). Each item is rated on a 5-point Likert scale ranging from 0 (*does not describe me*) to 4 (*describes me very well*). The Chinese version of the Interpersonal Reactivity Index (IRI-C) [31], was revised and normalized for a Chinese sample to assess empathy (*Likert 5-point*, from 0 to 4) [32]. This scale version is widely used in Chinese culture [32, 54, 55]. In the current study, the Cronbach’s α value for the four subscales (i.e., EC, PT, FS, and PD) was .51, .59, .70, and .62 respectively, which are consistent with values in the original English and Chinese versions of the IRI [30, 31, 40].

*Self-esteem*. The self-esteem was measured by Self-Esteem Scale (SES). The SES, developed by Rosenberg [56] originally, is a 10-item scale measuring global feelings of self-worth or self-regard, which is currently the most widely used self-esteem measure of this construct and has a good reliability estimate (Cronbach’s *α* = 0.84). Each item is rated on a 5-point Likert scale ranging from 1 (*strongly disagree*) to 5 (*strongly agree*), and higher scores on the SES indicate a higher level of an individual’s self-esteem. The Chinese version of the SES (C-SES) is widely used in Chinese culture [57–59], and the internal consistency coefficient of the C-SES was .89 in the present study which is consistent with the original version [56].

*The interpersonal relationships*. The interpersonal relationships were assessed with Basic Psychological Needs Scale (BPNS). The BPNS consists of 21 items and assesses an individual’s need satisfaction based on the three subscales of competency, autonomy, and relatedness [60]. Each item is rated on a 5-point Likert scale ranging from 1 (no agreement) to 5 (much agreement). The Chinese version of the Basic Psychological Needs Measurement Scale is widely used in China [61], with higher scores indicating greater needs. According to the purposes of the study, the subscale of relatedness was used in the current study to assess the quality of an individual’s relationships. In the current study, the internal consistency coefficient of the subscale of relatedness was .79.

*The prosocial behavior*. The prosocial behavior was assessed Prosocial Tendencies Measure (PTM). The PTM with 6 subscales (i.e., compliant, public, anonymous, dire, emotional, and altruism) was developed by Carlo et al. [62]. It consists of 28 items and is rated on a 5-point Likert scale ranging from 1 (*does not describe me at all*) to 5 (*describes me greatly*). A higher degree of prosocial behavior will be if an individual gets higher scores. Kou et al. [63] have established that the Chinese version of PTM has good construct and criterion-related validities. In the current study, the subscale of altruism was selected to measure prosocial behavior, and the internal consistency coefficient was .78.

#### Procedure

The study was conducted through a web-based survey via a Chinese survey website (https://www.wjx.cn). Advertisements were disseminated through popular online social media sites (i.e., Wechat). Participants first provided consent and then demographic information, and then completed the survey that consisted of various questionnaires including the ECQ-Chinese. The survey could not be submitted if any of the questions had not been answered, consistent with other published empathy studies [44]. Participants took approximately 10 minutes to complete the survey. The research procedure for this study conformed to the ethical standards of the 1964 Declaration of Helsinki. The Zhejiang Normal University Review Board approved the research procedures, and each participant signed an informed consent form before participating through an online survey mentioned above (e.g., "the responses to questionnaires would be anonymous and confidential which would be used only for academic research" and "you were at liberty to quit at any time").

#### Data analysis

First, SPSS (version 23.0) was used for item analysis, assessing the quality of items, which tests the difference of each item between the highest-scoring group and lowest-scoring group, and to examine the correlation coefficient between each item score and the total score. Second, exploratory factor analysis and confirmatory factor analysis were carried out to test the construct validity of ECQ-Chinese. Third, criterion validity analysis and reliability were used to examine the content validity and reliability (e.g., internal reliability, split-half reliability, and test-retest reliability). The full data for this study and subsequent studies are available on the Open Science Framework (https://osf.io/5cd7q/).

## Results

### Item analysis

Sample 1 was ordered by the ECQ total score from low to high. It is customary in psychometrics to refer to the top 27% of the sample as the high subgroup and the bottom 27% as the low subgroup, such as Zhang et al. [64], and Gao et al. [65]. The first 27% were chosen as the highest-scoring group, and the last 27% were chosen as the lowest-scoring group [66]. Kolmogorov-Smirnova and Shapiro-Wilk test found that it did not satisfy a normal distribution which is the assumption of the parametric test [67]. Therefore, a non-parametric independent sample test (Mann-Whitney U test) was carried out to compare the two groups for each item and the results showed that the difference between the highest-scoring group and lowest-scoring group was significant for each item (all *p’s* < .001). Next, the correlation coefficient between each item score and the total score was examined and the results showed that each correlation coefficient (range = .20 - .54) was significant for all items (all *p’s* < .001). Results indicated that the degree of differentiation between each item was good, and so all the items in the questionnaire were retained (see Table 1).

**Table 1:** Item analysis of ECQ-Chinese and the factor loading of EFA.

Item	*U* (*10^3^)	*r*	Loading	Item	*U* (*10^3^)	*r*	Loading	Item	*U* (*10^3^)	*r*	Loading
T1	7.73***	.23***	0.49	T10	6.17***	.31***	0.41	T19	5.32***	.52***	0.45
T2	6.28***	.32***	0.54	T11	4.55***	.45***	0.47	T20	5.60***	.42***	0.56
T3	6.72***	.32***	0.52	T12	5.85***	.37***	0.50	T21	6.93***	.20***	0.60
T4	5.40***	.44***	0.47	T13	8.02***	.19***	0.30	T22	7.42***	.22***	0.61
T5	5.13***	.50***	0.51	T14	5.22***	.48***	0.50	T23	5.68***	.37***	0.53
T6	4.56***	.48***	0.57	T15	6.62***	.28***	0.52	T24	5.49***	.44***	0.56
T7	6.78***	.48***	0.58	T16	6.06***	.34***	0.47	T25	5.82***	.44***	0.59
T8	5.42***	.46***	0.52	T17	5.39***	.44***	0.47	T26	5.57***	.40***	0.33
T9	4.88***	.48***	0.50	T18	6.21***	.30***	0.40	T27	6.92***	.31***	0.48

Note.

***p<0.001, *r* refers to Pearson’s coefficient between each item score and the total score; *U* refers to the non-parametric independent sample test (*Mann-Whitney U test*) between the highest-scoring group and lowest-scoring group; Loading refers to the loadings of the exploratory factor analysis.

### Exploratory Factor Analysis (EFA)

**Fig 1:**
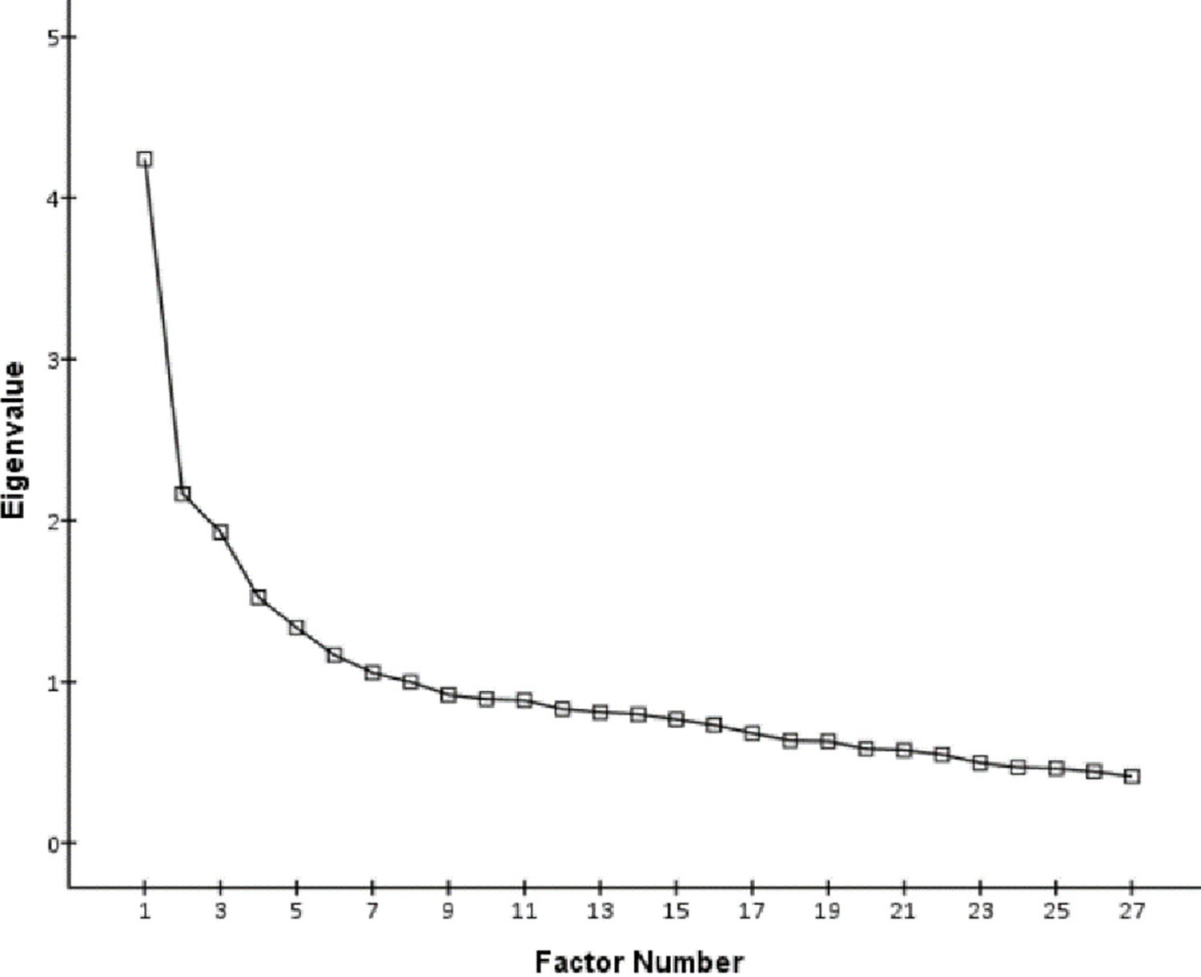
Scree plot for the ECQ- Chinese scale (sample 1, *n* = 538).

An exploratory factor analysis (maximum likelihood method: MLM) with a varimax (orthogonal) rotation method was conducted to determine the factor structure of the 27 items of ECQ-Chinese. The value of the *KMO* and Bartletts test of sphericity was 0.78, and *χ*^2^ = 2439.96, *df* = 351, *p* < .001, respectively, which indicated that the data was appropriate for exploratory factor analysis. Seven factors were extracted with an eigenvalue of more than 1, and the accumulative variance contribution accounted for 49.7%. Further, the scree plot (see Fig 1) indicated that "five" was the point of inflection, which explained 41.5% of the overall variation. Thus, the present results confirmed the factor structure of the original scale, and a further confirmatory factor analysis was conducted in Sample 3. Loadings of the exploratory factor analysis are presented in Table 1.

### Criterion validity analysis

Based on Sample 1, a criterion validity analysis was conducted and found that the participant’s scores on empathy, self-esteem, interpersonal relationship, and prosocial behavior were all significantly positively correlated with the total score of ECQ-Chinese and with the scores for each subscale (see Table 2). There was only one individual score that did not show a significant correlation coefficient which was between the subscale of affective response and self-esteem. Together, these results indicate that the ECQ-Chinese had good criterion validity.

**Table 2:** The criterion validity of the ECQ-Chinese.

criterion	Empathy	Self-esteem	Interpersonal relationship	Prosocial behavior
ECQ-CA	.19*** [.08, .28]	.29*** [.20, .37]	.25*** [.14, .35]	.14** [.05, .23]
ECQ-CD	.34*** [.26, .43]	.18*** [.07, .27]	.22*** [.12, .31]	.35*** [.26, 44]
ECQ-AA	.21***[.13, .29]	.35*** [.26, .43]	.47*** [.39, .54]	.17*** [.08, .26]
ECQ-AD	.40*** [.32, .47]	.22*** [.12, .31]	.36*** [.28, .44]	.43*** [.35, .51]
ECQ-AR	.40*** [.32, .48]	-.03 [-.13, .06]	.11* [.02, .19]	.17*** [.09, .25]
ECQ-Total	.47*** [.40, .54]	.32*** [.22, .40]	.44*** [.36, .52]	.38*** [.30, .46]

Note.

*p < .05

**p < .01

***p < .001. CA = cognitive ability; CD = cognitive drive; AA = affective ability; AD = affective drive; AR = affective reactivity. 95% confidence intervals (*CI*) are shown in parentheses.

### Internal and split-half reliability of the ECQ-Chinese

The internal reliability (i.e., homogeneity reliability) and the split-half reliability of the ECQ-Chinese and the subscales were tested by Cronbach alpha coefficient and Pearson’s correlation coefficients. The results showed that the ECQ-Chinese and the subscales have satisfactory internal consistencies (range = .49 - .76) with all *p’s* < .001, and satisfactory split-half reliability (range = .40 - .65) with all *p’s* < .001.

### Discussion

The present study aims to examine the quality of each item, criterion validity, internal and split-half reliability of the ECQ-Chinese. The results showed that the degree of differentiation between each item was good, which demonstrated the suitable quality of all the items. In addition, exploratory factor analysis found that the loadings of the EFA were good and it is appropriate to keep all the items. The findings for the internal consistencies and split-half reliability of the ECQ-Chinese demonstrated good reliability that met the psychometric requirements, and results of correlations with other relevant measures revealed good criterion validity. Together, the present results indicated the ECQ-Chinese has good content validity, structure validity, and reliability in a Chinse sample. These findings are consistent with those reported in previous studies of the ECQ in Western samples [34]. Together, the present results demonstrate that the ECQ-Chinese is a suitable measure for assessing empathy and its components in a Chinese sample. However, further test-retest reliability would provide even stronger evidence for the questionnaire stability in Chinese sample.

## Study 2 test-retest reliability analysis

### Materials and methods

#### Participants

In this study, 104 participants (77.88% female) were recruited from sample 1 to complete the ECQ-Chinese to investigate test-retest reliability three weeks after completing the initial survey including the ECQ-Chinese. The mean age for the sample was 19.65 with an *SD* ± 1.20 years.

#### Measures, procedure, and data analysis

The measures included the ECQ-Chinese and the same demographic information collected in Study 1, and the procedures in this study were the same as in Study 1. SPSS (version 23.0) was used for Test-retest reliability analysis (e.g., Pearson correlation coefficient).

### Results

#### Test-retest reliability analysis

The test-retest reliability analysis revealed that the Pearson correlation coefficients ranged from .51 to .70 and all were significant (all *p*’s < .001), which showed that the ECQ-Chinese has good test-retest reliability (see Table 3).

**Table 3:** The reliability of the ECQ-Chinese and the subscales.

Reliability	ECQ-CA	ECQ-CD	ECQ-AA	ECQ-AD	ECQ-AR	ECQ
Internal reliability	.61	.49	.54	.49	.53	.76
Split-half reliability	.61	.46	.39	.40	.55	.75
Test-retest reliability	.65*** [.50, .76]	.60*** [.44, .71]	.50*** [.34, .65]	.64*** [.49, .75]	.58*** [.44, .70]	.70*** [.56, .80]

Note.

***p < .001. Cronbach alpha coefficient (i.e., Internal reliability) and Pearson’s correlation coefficients (i.e., Split-half reliability and Test-retest reliability) are shown in the above table. 95% confidence intervals (CI) are shown in parentheses.

### Discussion

The present study aims to examine the test-retest reliability of the ECQ-Chinese. The results found a high consistency of the ECQ-Chinese when completed multiple times, as shown by the Pearson correlation coefficients reported here. The values of the test-retest reliability in this study ranged from .51 to .70 and all were significant (all *p*’s < .001), which were moderate and acceptable, and comparable to the test-retest reliability of the original scale [34]. However, a further confirmatory factor analysis would provide even stronger evidence for the construct validity of the ECQ-Chinese, and this was carried out in Study 3.

## Study 3 confirmatory factor analysis

### Materials and methods

#### Participants

The undergraduate samples were once again recruited from Zhejiang Normal University and Zhejiang Sci-tech University in Zhejiang province. The recruitment procedure was identical to Study 1. In this study, 335 undergraduate students (Sample 3) were recruited for confirmatory factor analysis of the ECQ-Chinese. Within this sample, 11 participants were excluded from the data screening process because they chose the same option on all the scales, which showed they were not suitably engaged in the survey. The final sample included 324 undergraduates (45.99% female), with their topics of study including a wide variety of areas such as pedagogy, science, literature, engineering, philosophy, etc. The mean age was 18.20 with an *SD* ± 1.03 years.

#### Measures, procedure, and data analysis

The measures included the ECQ-Chinese and the same demographic information as in Study 1, and the procedures in study 3 were the same as in Studies 1 and 2. Data treatment of confirmatory factor analysis (CFA) was performed using Mplus (version7.4), and the other statistical analyses were conducted using SPSS (version 23.0). Furthermore, the goodness-of-fit of confirmatory factor analysis (CFA) was evaluated by multiple criteria: chi-square (*χ*^2^), the root mean square error of approximation (RMSEA), the comparative fit index (CFI), the nonnormed fit index (NNFI/TLI), and the standardized root mean square residual (SRMR). According to the recommendations within this field, the following criteria indicate good model fit: *χ*^2^/*df* < 3, RMSEA < 0.08, CFI > 0.85, TLI > 0.85, SRMR < 0.08 [68–70].

### Results

#### Confirmatory Factor Analysis (CFA)

**Fig 2:**
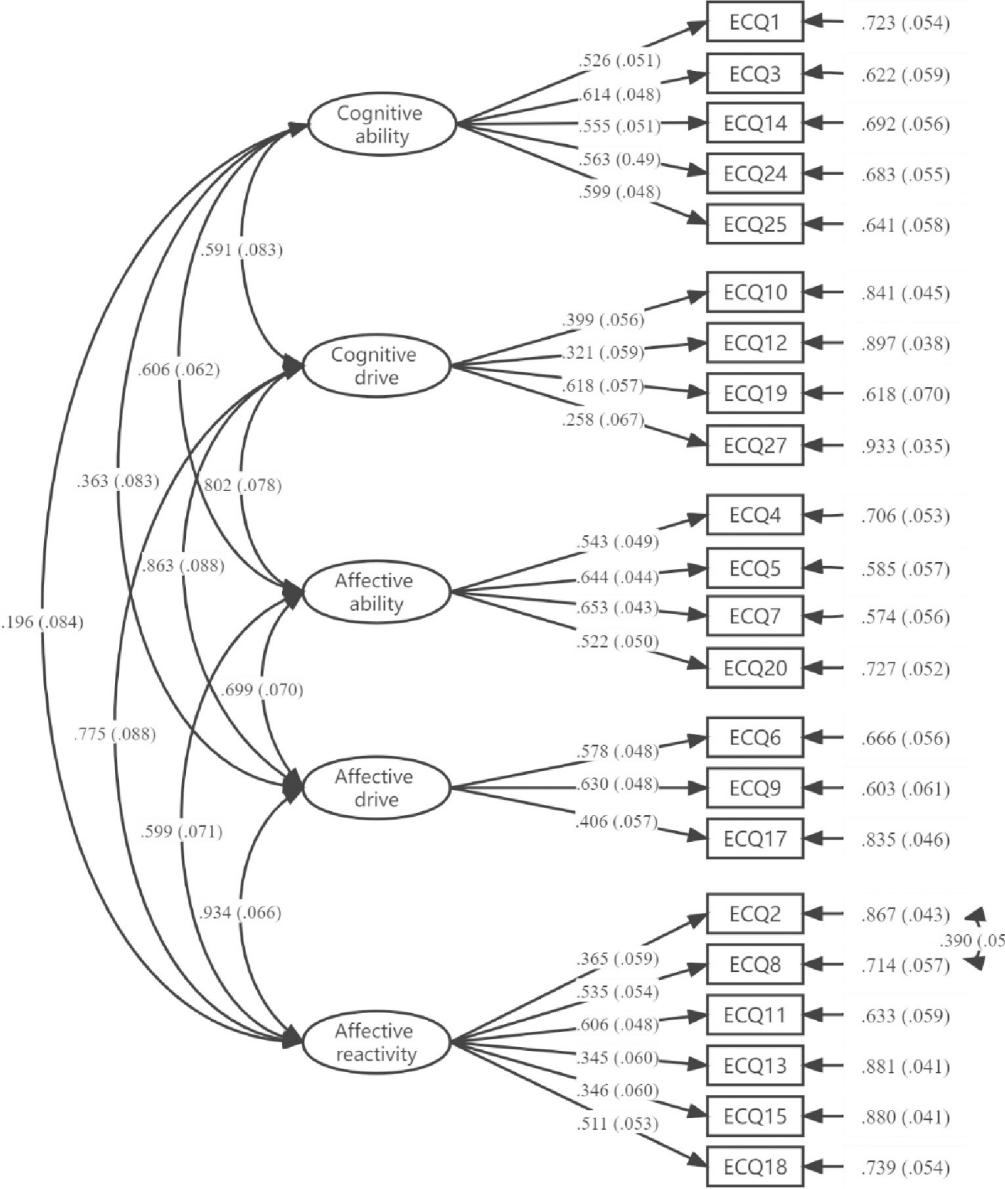
Confirmatory factor analysis standardized estimates of the ECQ-Chinese scale (sample 3, *n* = 324).

A confirmatory factor analysis was run on sample 3 (*n* =  324) using the five-factor structure obtained in the original questionnaire. Considering the theoretical and practical significance of items, the model was modified according to the modification index (MI). The items "When someone seems upset, I am usually uninterested and unaffected by their emotions" (t2) and "Others’ emotions do not motivate my mood" (t8) have a similar meaning about one’s mood not being affected by the mood of others. Thus, the residuals between t2 and t8 were allowed to co-vary. The results from the CFA showed that the factor loadings are relatively low in some items (less than .30) (see *Appendix B in S1 Appendix*), such as t16, t21, t22, t23, and t26. Based on this, we culled these items and re-conducted a confirmatory factor analysis, the results showed that the measurement model met the criteria for a good data-model fit (*χ*^2^/*df* = 1.99, RMSEA = 0.06, 90% *CI* [0.05, 0.06], CFI = 0.86, TLI = 0.83, SRMR = 0.06) [68–70], and factor loadings are shown in Fig 2. Thus, the final version of the ECQ-Chinese contains 22 items in total, and the composite reliability, convergence validity and discriminate validity were shown in Table 4.

**Table 4:** Composite reliability, convergence validity and discriminate validity.

DIM	Item reliability	Composite reliability	Convergence validity	Discriminate Validity				
	STD.LOADING	CR	AVE	CA	CD	AA	AD	AR
**CA**	0.53–0.61	0.71	0.33	**0.57**				
**CD**	0.26–0.62	0.44	0.18	0.35	**0.42**			
**AA**	0.52–0.65	0.68	0.35	0.42	0.43	**0.59**		
**AD**	0.41–0.58	0.55	0.30	0.25	0.42	0.42	**0.55**	
**AR**	0.35–0.61	0.61	0.21	0.12	0.33	0.37	0.47	**0.46**

The square root of AVE for each subscale is in the diagonals and bold, and the lower triangle is the Pearson’s correlation coefficient.

### Discussion

Study 3 aims to examine the construct validity of the ECQ-Chinese. Consistent with the original study, we adopted a five-factor measurement structure model which included the subscales of cognitive ability, cognitive drive, affective ability, affective drive, and affective reactivity. Based on the results of the first CFA, we culled 5 items that the factor loadings were relatively low, and re-conducted a confirmatory factor analysis. The results showed that the measurement model (i.e., including 22 items) met the criteria for a good data-model fit. However, the factor loading of some items was on the lower side, such as t12 and t27. One possible explanation for this is cultural and language differences between the UK and China [39, 44]. Subjects in different cultural backgrounds may have slightly different understandings of the meaning for some items, and different situations and behaviors may have slightly different contexts across cultures. However, the present results revealed the ECQ-Chinese scale (22 items) to have good construct validity.

## General discussion

The current study translated and revised a Mandarin Chinese version of the Empathy Components Questionnaire (ECQ-Chinese), which was validated in three Chinese samples. The results supported the five component model of the original questionnaire in the ECQ-Chinese version and retained 22 items in Chinese culture. The present findings verified that the ECQ-Chinese has good reliability and validity, and thus provides a new tool suitable for measuring empathy and its components in the context of Chinese culture. The validation of the ECQ-Chinese provides at least three theoretical and practical implications.

### Exploring deficits in the components of empathy

Empathy is considered to be a critical process within interpersonal interactions [47, 48]. These include situations such as collaborations and prosocial behaviors [49], as well as inhibiting aggressive behavior [71]. Conversely, deficits in empathy have been associated with a range of conditions [8], such as autism spectrum disorder (ASD) [10, 72], and psychopathy [73–75]. Previous clinical research has mainly focused on investigating empathic ability, including previous empathy research in China. However, as outlined above empathic ability is just one of the key components of empathy within a wider framework. It has been proposed that psychopathy may be characterized by impaired affective empathy alongside intact or even superior cognitive empathy, while ASD is characterized by impaired cognitive empathy and intact affective empathy [76, 77]. There may also be differences between ASD and psychopathy in the ability versus the motivation towards empathy [26]. Since the population of China is approximately 1.4 billion and prevalence rates are reported to be approximately 1% for ASD [78], and for psychopathy [79], this suggests there are about 14 million autistics and people with psychopathy in China. The ECQ-Chinese provides a quick and easy measure to investigate empathy differences in Chinese samples of autistics and those with psychopathy to test ideas about patterns and differences in empathic components between these conditions.

### Cross-cultural empathy research

While some studies have reported differences in subscales of the IRI between Western and Chinese samples [38, 41, 42], other studies have reported contradictory findings to these [37, 43, 44]. One of the reasons for this might be some of the subscales of the IRI (e.g. the fantasy and personal distress subscale) are not empathy-specific measures and may index broader processes than empathy [10]. Another reason may be that previous assessments of empathy used in cross-cultural studies (e.g. the IRI, EQ, and MET) do not index all the components of empathy in current theoretical frameworks, which include both cognitive and affective empathy and also ability and motivation within each of these domains. Therefore, the contradictory cross-cultural findings of empathy in Chinese samples to date may be due to measurement issues. Further cross-cultural research would benefit from an assessment tool that indexed the wider components of empathy including both empathic ability and motivation. The development and validation of the ECQ-Chinese questionnaire help to fill this gap by providing a tool to facilitate further cross-cultural studies of empathy and its components in a systematic way using the same comprehensive empathy measure for comparisons between studies.

### Testing effects of empathic motivation interventions

Furthermore, the ECQ-Chinese provides a tool for measuring the effectiveness of empathic motivation interventions. Empathy training is a popular topic and most participants report being interested in improving their empathic processes, and positive correlations are found between participants’ willingness and their development of empathy [80]. Most current empathy interventions focus on the training of empathic ability [81]. For example, experience-based empathy training aims to encourage perceivers to experience the target’s inner feelings, while expressive-based empathy training aims to encourage perceivers to recognize the target’s internal state and make appropriate emotional responses [82]. Specific interventions include role-playing, virtual reality [83], meditation [84, 85]. However, previous empathy interventions have not shown effective results in terms of greater empathy scores for participants after the empathy training compared to their empathy scores before the training. For instance, a meta-analysis by Waller et al. [86] reported that the effect of empathy training was only moderate *(Hedges’g* = 0.51). The reason for this finding may be that most of the current empathy interventions focus on the cultivation of empathic ability, and ignore empathic motivation. Therefore, empathy intervention should broaden the ideas and methods to widen the framework of empathy "ability" training to include aspects that target "motivation" as well. The ECQ-Chinese provides a new useful tool for testing the effect of the intervention on empathic motivation in the context of Chinese culture.

### The limitations of the current study

There are some limitations of the current validation study of the ECQ-Chinese that should be noted. First, the present study employed only self-report measures, which might be susceptible to response bias (e.g., social desirability). Second, based on the size of the samples and the fact the participants were all university students, the results may not be representative of the general population. Further research is needed including larger and more representative samples including both typically developed participants and those with clinical conditions. Finally, the ratio of gender was not balanced in this sample because the sampling is mainly from normal universities where there are more girls than boys in China. Future research should pay more attention to the balanced gender ratio of samples. Despite these limitations, the present study revealed results to validate the ECQ-Chinese for measuring affective and cognitive empathy as well as empathic ability and motivation in China, and a tool for cross-cultural studies of empathy in Chinese samples compared to samples from other countries and cultures.

## Supporting information

S1 Appendix (DOCX)
