# Supplementary material for: The validation of a Mandarin version of the Empathy Components Questionnaire (ECQ-Chinese) in Chinese samples
Source: PLoS One. 2023 Jan 26;18(1):e0275903. doi: 10.1371/journal.pone.0275903 (PMC9879452; doi:10.1371/journal.pone.0275903)
Supplement: S1 Appendix — (DOCX) [file pone.0275903.s001.docx]

***Appendix A*** (Copyright 2017 by Ashwin. Reprinted with permission)

T1 I am usually successful in judging if someone says one thing but means another.

T2 When someone seems upset, I am usually uninterested and unaffected by their emotions.

T3 I am not very good at predicting what other people will do.

T4 My friends often tell me intimate things about themselves as I am very helpful.

T5 I am good at responding to other people’s feelings.

T6 I am not interested in protecting others, even if I know they are being lied to.

T7 I am not very good at helping others deal with their feelings.

T8 Others’ emotions do not motivate my mood.

T9 I have a desire to help other people.

T10 When talking with others, I am not very interested in what they might be thinking.

T11 I feel pity for people I see being bullied.

T12 I strive to see how it would feel to be in someone else’s situation before criticizing them.

T13 I avoid getting emotionally involved with a friend’s problems.

T14 I do well at noticing when one of my friends is uncomfortable.

T15 I like to know what happens to others.

T16 I am uninterested in putting myself in another’s shoes if I am upset with them.

T17 When I do things, I like to take others’ feelings into account.

T18 I am not always interested in sharing others’ happiness.

T19 I like trying to understand what might be going through my friends’ minds.

T20 I am poor at sharing emotions with others.

T21 When someone is crying, I tend to become very upset myself.

T22 I don’t intuitively tune into how others feel.

T23 I avoid thinking how my friends will respond before I do something.

T24 I am not very good at noticing if someone is hiding their emotions.

T25 During a conversation, I’m not very good at figuring out what others might want to talk about.

T26 I am good at sensing whether or not I am interrupting a conversation.

T27 I take an interest in looking at both sides to every argument.

***Appendix B***

**
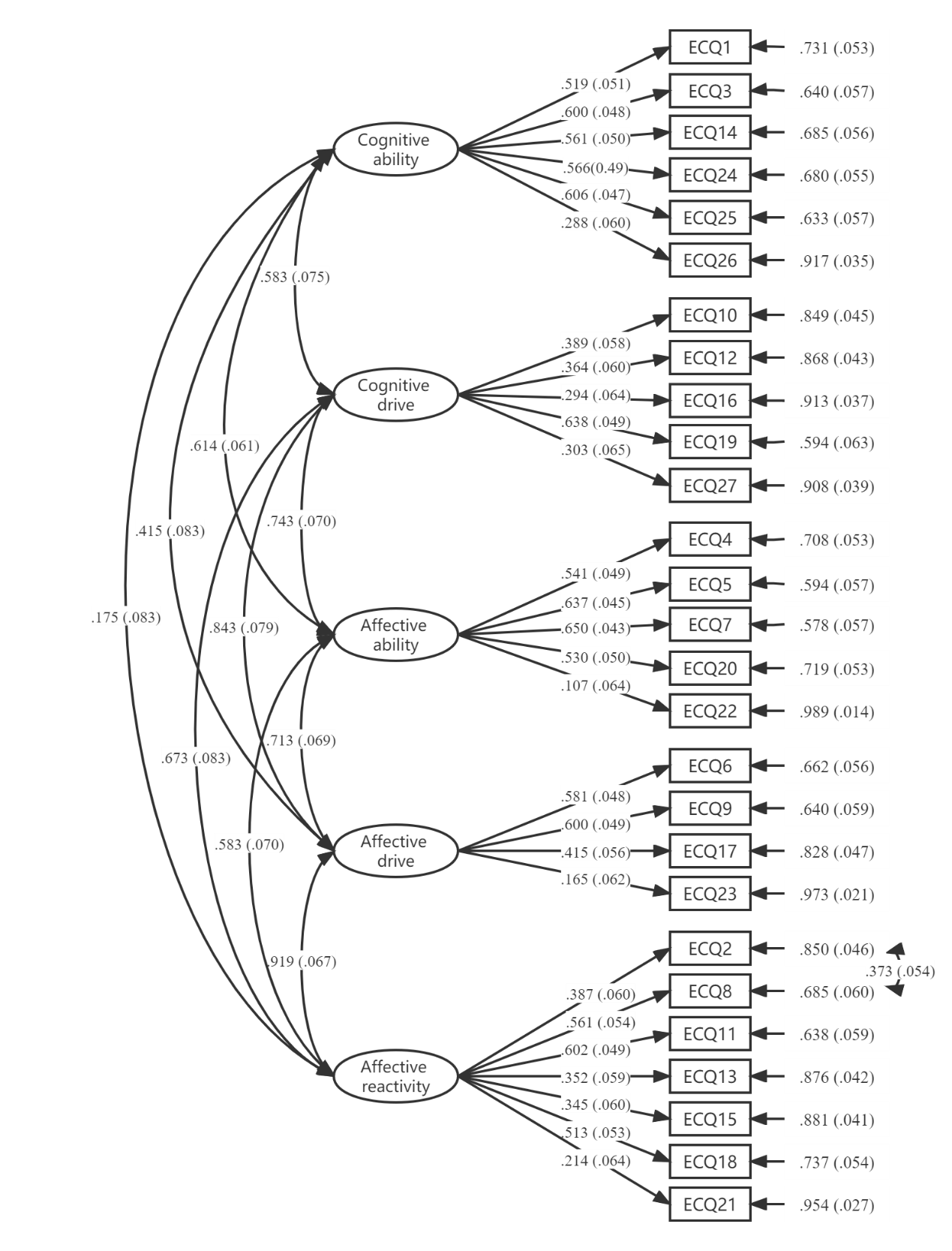
**
